# Draft Genome Sequence of Marine Bacterium Streptomyces sp. Strain CNQ431, a Producer of the Cytokine Inhibitor Splenocin

**DOI:** 10.1128/genomeA.01383-14

**Published:** 2015-01-22

**Authors:** Mingjia Yu, Zheng Dai, Xudong Qu, Xu Gao

**Affiliations:** a Department of Preclinical Medicine, Harbin Medical University, Harbin, China; b School of Pharmaceutical Science, Wuhan University, Wuhan, China

## Abstract

Currently, corticosteroids are the most potent anti-inflammatory drugs on the market. Here, we announce the draft genome sequence of the marine-derived Streptomyces sp. strain CNQ431, which produces cytokine inhibitors, termed splenocins, which display potent suppression of cytokine production at a comparable level to that of corticosteroids. The genome is approximately 498,750 bp with 72.03% G+C content.

## GENOME ANNOUNCEMENT

With the increasing rate of morbidity and mortality caused by chronic obstructive respiratory disorders, such as asthma, these diseases rank as the fourth leading causes of death worldwide (1). In the pathogenesis of chronic asthma, one of the early cellular events is the stimulation of T_H_2 lymphocytes by antigen-presenting cells (APCs), which can secrete a number of cytokines, eventually leading to the asthmatic phenotype (2, 3). Therefore, the compounds that suppress the production of multiple T_H_2 cytokines might possess therapeutic potential for the treatment of asthma (4, 5). However, there have been few novel anti-inflammatory drug classes developed to treat chronic asthma other than the corticosteroids (6). Later, by focusing more on the marine microbial secondary metabolites, Fenical and Jensen (7) discovered that splenocins from this strain have immunosuppressive effects on both the APCs and T_H_2 cells, causing a potent inhibition of cytokine production.

Streptomyces sp. strain CNQ431 was isolated from oceanic bottom sediments off the coast of the Scripps Institution of Oceanography in La Jolla, CA (8). The genome was sequenced using Illumina technology, with an average sequencing coverage of approximately 110×. The results of the genome sequence were assembled in a paired-end (PE) DNA library and a mate-pair (MP) DNA library using the TruSeq DNA sample prep kit-set A and TruSeq PE cluster kit, Illumina automatic sequencing system, and the software Velvet 1.2.03 (9, 10). The draft genomic DNA sequences of the PE and MP DNA libraries were 12,046,184 and 4,258,782 total bases with 100-bp sequencing length, respectively. The assembly results of the genomic sequences using the Velvet 1.2.03 software showed that it is composed of 295 contigs with 7,037,644 bp and 32 scaffolds with 7,077,925 bp, which both have about 72% G+C content. Next, autoannotation was performed using the software Glimmer with the contigs mentioned above, as well as sequence alignment with NCBI, yielding 6,197 predicted genes, of which 4,837 genes have definite biological function, 37 tRNAs, and 2 rRNAs.

As anticipated based on its 16S rRNA-based phylogeny, the available genes in CNQ431 are most similar to the organism classification of Streptomyces sp. strain S4. By using the KEGG resource for deciphering the genome to illustrate the function of genes and by using the CDD database to make COG function classifications (11), we announce that 2,054 open reading frames (ORFs) have KEGG orthologs, 4,140 ORFs have COG categories, and all the homologous proteins that were used to match these functional genes derive from 153 species, with the strain Streptomyces albus J1074 having the highest ratio rank (12).

The announcement of the draft genome sequence of Streptomyces sp. CNQ431 will provide a comprehensive understanding and further investigation of this genus; we hope to find the specific biological functional genes that act as anti-inflammatory agents and play a pivotal part in the suppression of cytokine production by APCs and T cells and that are beneficial to the treatment of chronic asthma disease.

### Nucleotide sequence accession number.

The whole-genome sequence of the Streptomyces sp. CNQ431 strain has been deposited in the GenBank database under the accession no. JTCK00000000.
